# Chronic Laryngitis

**Published:** 1908-07-04

**Authors:** Harold Barwell

**Affiliations:** Surgeon for Diseases of the Throat to St. George's Hospital; Laryngologist to the Mount Vernon Hospital for Diseases of the Chest, etc.


### Hospital Clinics.

### CHRONIC LARYNGITIS.

By HAROLD BARWELL, M.B., F.R.C.S., Surgeon for Diseases of the Throat to St. George's Hospital; Laryngologist to the Mount Vernon Hospital for Diseases of the Chest, etc.

Chronic laryngitis is an important unit in the long list of so-called "minor ailments," which, though they do not shorten life, yet cause a vast amount of discomfort, and even interfere seriously with the sufferer's occupation and means of livelihood. It must ever be remembered that the hoarseness, which is its principal symptom, may be due to many other causes, and that it is impossible to diagnose simple catarrh of the larynx without laryngoscopic examination. To treat a papilloma of the vocal cord without examination by a prolonged course of rest and sprays is an error which will not redound to the credit of the physician, but to treat an early epithelioma in the same way is a disaster.

### Symptoms.

The constant and obvious symptom is impairment of the voice; there is nothing characteristic about the kind of hoarseness found in this affection, for the voice may be rough, husky, weak, or even aphonic, and sometimes it varies suddenly in tone like that of a boy whose voice is "breaking." It is easily tired, and is usually worse after use and in the evening, but sometimes, in the dry forms of laryngitis associated with mouth-breathing and nasal disease, it is at its worst in the early morning and improves after breakfast. The patient usually also complains of some local discomfort, a sense of dryness, excoriation or tickling, or of a feeling of swelling. There is often an irritable cough, but, unless the trachea and bronchi are involved, there is little expectoration.

### Objective Appearances.

The laryngoscopic appearance in cases of chronic catarrh is very variable, and by no means always strictly proportionate to the degree of vocal impairment. Much of the hoarseness is due to imperfect action of the laryngeal muscles, caused partly by spread of the inflammation to the muscle-tissues and partly, perhaps, by stiffness or rigidity of the hyperæmic mucous membrane interfering with the easy action and delicate adjustment necessary to produce a good voice. The general muscular and nervous tone of the individual is also largely concerned, and many patients, more particularly elderly women, become completely aphonic with a very slight catarrh, whereas in other cases the voice remains practically unaffected even though the vocal cords be markedly reddened. Indeed, in most cases of the so-called "functional aphonia," or adductor paresis, a certain amount of catarrhal laryngitis is present to initiate the attack.

The larynx is variously reddened, and this is naturally most visible on the normally white vocal cords; the hyperæmia is generally less bright and more unevenly distributed than is the case in acute laryngitis. In the slightest cases the cords have merely lost their normal pearly lustre and are dirty-white, grey, or pink; the vocal processes are often prominent, and may be reddened, or they may show up white against the redness of the cords. Enlarged vessels may often be seen upon the surface of the cords, and they are frequently slightly thickened so that the sharp inner edge has given place to a more rounded contour. This swelling may be greatest about the centre of the cord, making the line of its edge convex instead of straight, and giving to the cord a slightly "barrel-shaped" appearance; when this is the case the two cords touch at their centres during phonation, instead of leaving the normal narrow slit-like interval between them, and, as this is also the point of maximum vibration, a minute corn or "singer's nodule" may form at this spot. These little excrescences are practically confined to this the mid-point of the vocal cord; it has often been described as the junction of the anterior and middle thirds of the cord, but this is due to the error of including the cartilaginous glottis in the measurement. This point is also the commonest site, or "seat of election," for all innocent tumours of the cord; there is little doubt that most of these are the result of irritation and, indeed, no distinct line of demarcation can be drawn between the fibromata, cysts, and papillomata which occur at the seat of election, and simple inflammatory thickenings. During phonation, also, a globule of mucus frequently forms at the centre of the cord, where it may, at first sight, be readily mistaken for a singer's nodule. Collections of mucus may often be seen in the larynx, and strings of sticky secretion sometimes stretch across the glottis; this is characteristic of chronic, as distinguished from acute, catarrh, for in the latter affection the secretion is not viscid and does not become aggregated into masses. The epiglottis is frequently injected, and enlarged vessels are visible on its surface; the mucous membrane is stretched tightly over the edge of the cartilage, so that the capillaries cannot become engorged, and the yellow margin of the organ forms a vivid contrast to its reddened surface. Rarely the mucous glands may be enlarged to form numerous small projections, especially over the arytenoids and aryteno-epiglottidean folds, where such glands are most numerous; the term "granular laryngitis" has been given to this condition. There is also a collection of glandular tissue in the anterior commissure immediately below the level of the cords, and this may become swollen and form a small red mass which prevents their proper apposition. This is quite an uncommon occurrence, but it deserves mention because it may produce marked hoarseness, and, unless it be thought of, may be readily overlooked, for the anterior commissure is the most difficult part of the larynx to inspect.

*Laryngitis sicca* is a form generally, but not invariably, associated with rhinitis sicca or atrophic rhinitis, and with the typical dry glazed pharynx of dry pharyngitis. The mucous membrane of the larynx is also dry and glazed, but to a less extent, and small brown crusts are apt to form and to adhere to the cords and interarytenoid region; these may give rise to hæmorrhage when detached by coughing. This variety passes imperceptibly into the more marked form of *atrophic laryngitis*, which is practically always associated with atrophic rhinitis; large green or black fœtid crusts form, and may even extend down the trachea and cause suffocation. Ulceration takes place beneath the crusts, and large sprouting granulations may be formed. The affection is fortunately quite rare; although atrophic rhinitis is common enough, it is usually associated only with a simple or dry chronic laryngitis, and crusting very rarely occurs within the larynx itself.

A slight swelling or relaxation of the mucous membrane in the posterior commissure is the rule, and, as I have mentioned, the vocal processes are often somewhat prominent; from this all gradations may be met with up to the most extensive pachydermatous thickening. *Pachydermia laryngis*, indeed, is probably not so much a separate and distinct disease as a development of chronic laryngitis, though the nature and causation of this important condition is still something of a mystery. From a pathological point of view, the little thickenings of the vocal cords, known as singer's nodules, may be classified under pachydermia, though they are found in a very different class of patient. The essential process is a thickening of the epithelial and subepithelial tissues, and the disease affects principally the vocal processes, cartilaginous glottis and interarytenoid space. In the latter region the earliest appearance is that of a slight thickening as if the part were covered with a thin grey pellicle, which wrinkles when the arytenoids are brought together, but is smoothed out again on wide abduction. Then these wrinkles become permanent, and the swelling increases so as to fill the angle between the inner surface of the arytenoid cartilage and the posterior wall of the larynx; the thickening is more or less symmetrical, and usually leaves in the middle line of the posterior commissure a characteristic fissure or depression; but this is not invariably the case, for pachydermatous thickening may form a smooth median swelling on the posterior wall. The surface may be smooth or evenly crenated like a cock's comb. On each vocal process a circumscribed swelling appears, and at the apex of the prominence a small depression or umbilication develops, either as the result of the pressure of the opposite vocal process or because the mucous membrane is more firmly adherent to the subjacent cartilage at this point. The cords come into more thorough apposition and, consequently, the voice remains better than might be expected from the size of the projections; the reason for this is that the prominences consist of a series of ridges lying one above the other, and that a certain amount of vertical displacement occurs so that the projection on the one side fits into the depression on the other; in doing this the left vocal process usually lies at a lower level than the right. Erosions, and even definite ulcers, sometimes form, and generally on the upper surface of the left and on the lower surface of the right vocal process. It need hardly be said that ulceration does not occur in simple chronic laryngitis.

### Ætiology.

Chronic laryngitis is often the result of repeated or persistent acute catarrh, but the inflammation may also be chronic from the outset. The question of causation is of considerable importance, for it is only by discovering and treating the cause that we can expect to obtain satisfactory results. The chief factors in its causation may be classified as follows:—(1) Nasal obstructions and suppurations; the latter act by direct irritation of the pus which reaches the larynx, and by spread of the inflammation by continuity; the former owe their effect to mouth-breathing, whereby dry, cold, and dusty air is inhaled into the larynx. Atrophic rhinitis and rhinitis sicca must also be mentioned as powerful causes; atrophic rhinitis acts in both these ways, by reason of the irritation of the discharges, and of the unfiltered air, and by the gradual extension of the atrophic condition to the pharynx, and so into the larynx; but, as stated above, true atrophic laryngitis is a decidedly rare condition, and usually the larynx is only affected by a simple chronic laryngitis. In voice-users nasal obstruction acts in yet another way—namely, by destroying the resonance of the voice, and so causing a greater effort in voice-production. (2) Working in stuffy and dusty rooms; a good instance of this may be observed in the effect of chalk from the black-board on the voice of a school-teacher. In such subjects a chronic laryngitis may often be improved to quite a remarkable extent by substituting a damp sponge for the usual dry cloth which is well-named a "duster." (3) Dyspepsia, especially when combined with the abuse of alcohol or tobacco; the foul tongue and irritable inflamed pharynx of dyspepsia is well known, but the chronic intractable laryngitis which often accompanies these conditions is, perhaps, not so generally recognised. (4) Anæmia, rheumatism, and gout are also important factors; with regard to the former, I have already mentioned the considerable part which loss of muscular tone plays in producing the hoarseness which is the principal symptom of this affection, so that a slight catarrh, which might produce no symptom in a vigorous individual, may cause complete aphonia in an unhealthy and anæmic patient. Pulmonary phthisis is a frequent cause of simple chronic laryngitis, by reason of the debility, the frequent cough, and the irritation of the sputum. A persistent laryngitis in a phthisical patient will naturally cause great anxiety, for in its earliest stage tuberculous laryngitis may be quite indistinguishable from simple catarrh; yet in a large number of cases the lesion is purely catarrhal, and hoarseness and aphonia in a consumptive must not be considered diagnostic of tuberculous laryngitis. A certain degree of pachydermatous thickening is common in the larynges of consumptives; indeed Gougenheim has considered that pachydermia is usually due to tuberculosis; it is probable that the chronic irritation from cough and sputum, to which a consumptive's larynx is liable, is a sufficient cause of pachydermia without there being, of necessity, any invasion by the tubercle bacillus. (5) Overuse of the voice, and more particularly incorrect voice-production, are among the most important factors in the ætiology, especially as the treatment of chronic laryngitis is of the greatest importance among professional voice-users. The different methods of using and misusing the voice, and the various lesions which result, vary considerably in different occupations. In the interesting opening paper of a discussion before the British Medical Association, Dr. Middlemass Hunt * divides the forms of voice-use and the varieties of trouble which usually result as follows:—

1. Use of voice through its whole compass, at definite degrees of pitch, and usually for short periods of time—singers. Lesions: hyperæmia, muscle paresis.

2. Use of voice for prolonged periods, but with prolonged intervals of rest—preachers, public speakers, and actors. Lesions: chronic congestion, phonasthenia, neurotic disturbances.

3. Daily use of the voice for many hours at a stretch — school-teachers. Lesions: "singer's nodules," fibromata.

4. Violent use of the voice in unfavourable surroundings, and I may add, in the worst methods of production—hawkers, drill-sergeants, and auctioneers. Lesions: submucous hæmorrhage, rupture of muscle or ligament, pachydermia.

5. Use of voice at an unnatural pitch or in an unnatural manner—actors in certain *rôles*, choir-masters, and falsetto singers. Lesions: congestion, hypersecretion, and benign growths.

It must not be considered that this is in any way a hard and fast rule, or that it establishes a law, but, on the whole, I think that Middlemass Hunt's classification may be taken as a useful guide to what one may expect to find among the various kinds of voice-users. Certainly, "singer's nodules" are common among teachers, especially women, and are rarely seen in the throats of professional singers, whereas pachydermia is undoubtedly most often found in the hawker class. As to the causation of pachydermia, it is much commoner in men than in women, and is chiefly a disease of middle life. Most English laryngologists believe that alcoholism is the principal factor in its production; Moritz Schmidt considers that it occurs only in association with a dry rhino-pharyngitis, and is of the nature of a corn due to the frequent cough; whilst Gougenheim, as mentioned above, is of the opinion that it is essentially tuberculous.

### Diagnosis.

But little need be said on this subject. It must never be forgotten that, though catarrh may be the most frequent cause of hoarseness, other important affections are at first only manifest through this symptom, and that the diagnosis can only be safely made after excluding other diseases by means of the laryngoscope. If distinct ulceration be seen, the case may be anything except one of simple catarrh. Redness of one cord, or much more marked on one side, may be caused by syphilis, tuberculosis, or cancer, but is hardly ever due to catarrh alone.

* *British Medical Journal*, vol. ii. p. 1472, 1906.

Typical cases of pachydermia are easily recognised, especially by their firm and symmetrical appearance and by their chronic course; the umbilicated projections on the vocal processes are also very characteristic. The swollen and sometimes eroded vocal processes may suggest syphilis to the unwary, but the erosions are less well defined, and there is less hyperæmia and injection. The interarytenoid infiltration of tuberculosis may be very similar, and, as I have said, it is doubtful if some cases of pachydermia are not tuberculous; but the usual soft, translucent and irregular swelling of tuberculosis is very characteristic, and, if definite ulceration be seen, the diagnosis is at once decided.

### Treatment.

The detection and elimination of the casual factors are by far the most important part of treatment; it may be said at once that, if these cannot be removed or alleviated, the affection is most intractable. Pharyngitis and bronchitis are common concomitants, and must receive attention at the same time. A very minute lesion will damage the larynx for such exact action as is required in singing, and therefore no abnormality which the laryngoscope can detect may be found in the larynx of the professional patient who comes to you for advice, and the progress of the case must be judged by the effect on the voice rather than by any objective changes in the organ. With professional singers and speakers the damage is done to the voice not so much by overuse as by faulty production, provided always that the larynx is healthy to start with, and that there is no other physical cause, such as nasal obstruction, to account for the break-down. I have no time on this occasion to discuss the correct methods of voice-production, and I must confess that this is a difficult matter to deal with when advising a professional singer of standing, though hints will often be gratefully received by speakers. In brief, a perfect freedom and looseness of the muscles must be attained, and the resonance of the voice should be brought well forward into the nasal cavity. Holbrook Curtis' vocal exercises are of value in this connection; his little book* is well worth perusal by the doctor who has to treat one of these troublesome cases.

Locally a stimulating solution in a nebuliser may be ordered, such as oleum pini sylvestris, eucalyptus, or cubebs. A stringent application may be applied with a mop, but this is seldom advisable except when there are hypertrophic changes; chloride of zinc 15-30 grs. to the ounce, or silver nitrate 10-50 grs., are the drugs usually employed; the latter is the most effective and the most painful. The weaker solutions should be used first and the strength slowly increased; the applications must be made by the physician daily or every other day for a considerable time. Atrophic laryngitis demands the exhibition of expectorants and the use of an oily spray; in marked cases of pachydermia solid silver nitrate may be applied to the projections, or an alcoholic solution of salicylic acid, one to six per cent. Internally, small doses of potassium iodide or proto-iodide of mercury may be given, and, when the muscles act

* "Voice-building and Tone-placing."

badly, strychnine is indicated. In voice-users, and especially when the cords are dull and dry, expectorants are of value, such as a mixture of ammonium carbonate and squills or ipecacuanha; expectorant lozenges are convenient, and the trochisci acidi benzoici, trochisci ammonii chloridi, and trochisci cubebæ of the Throat Hospital Pharmacopœia are among the most useful. When possible, complete vocal rest is a most valuable therapeutic measure; in a professional voice-user, when the affection prevents him from following his occupation, it is advisable to order complete silence, and to persuade him to communicate his wants only by signs or with a slate and pencil.

